# Retraction: The CRAVE and ARGE scales for motivation states for physical activity and sedentarism: Brazilian Portuguese translation and single-item versions

**DOI:** 10.3389/fpsyg.2026.1946252

**Published:** 2026-07-30

**Authors:** Frontiers Editorial Office

The Journal retracts the article cited above.

Following publication, concerns were raised regarding the integrity of the Brazilian dataset used in Study 1 and, in part, in Study 3, specifically duplication of data rows, errors in the computation of derived variables, and incorrectly recorded values.

The first author acknowledges that the published Brazilian dataset was not the correct final version and contained substantial handling errors: duplicated response patterns, inclusion of participants below the intended minimum age criterion, incorrect BMI calculations, and incorrect application of scoring weights for the Godin–Shephard Leisure-Time Physical Activity Questionnaire. As a result, the effective Brazilian sample was reduced from the originally reported 1,168 entries to 541 valid participants. Re-analysis using the corrected Brazilian dataset no longer supports the claim that these data constitute Brazilian normative data. The article has therefore been retracted.

This retraction was approved by the Chief Editors of Frontiers in Psychology and the Chief Executive Editor of Frontiers.

Frontiers would like to thank Abel Brodeur, Kristjan Moore, Niklas Jakobsson and Bruno Barbarioli for contacting the journal regarding the published article.

